# Identifying Gene Networks Underlying the Neurobiology of Ethanol and Alcoholism

**DOI:** 10.35946/arcr.v34.3.05

**Published:** 2012

**Authors:** Aaron R. Wolen, Michael F. Miles

**Affiliations:** **Aaron R. Wolen, Ph.D.,***was a doctoral candidate in the Department of Human and Molecular Genetics, Virginia Commonwealth University, Richmond, Virginia.*; **Michael F. Miles, M.D., Ph.D.,***is a professor in the Departments of Pharmacology and Toxicology and Neurology, Virginia Commonwealth University, Richmond, Virginia.*

**Keywords:** Alcoholism, alcohol use disorders (AUDs), genetics, genetic basis of alcoholism, genetic technology, genetic association studies, quantitative trait loci (QTLs), genetic mapping, gene networks, genomes, genetical genomics, human studies, animal models

## Abstract

For complex disorders such as alcoholism, identifying the genes linked to these diseases and their specific roles is difficult. Traditional genetic approaches, such as genetic association studies (including genome-wide association studies) and analyses of quantitative trait loci (QTLs) in both humans and laboratory animals already have helped identify some candidate genes. However, because of technical obstacles, such as the small impact of any individual gene, these approaches only have limited effectiveness in identifying specific genes that contribute to complex diseases. The emerging field of systems biology, which allows for analyses of entire gene networks, may help researchers better elucidate the genetic basis of alcoholism, both in humans and in animal models. Such networks can be identified using approaches such as high-throughput molecular profiling (e.g., through microarray-based gene expression analyses) or strategies referred to as genetical genomics, such as the mapping of expression QTLs (eQTLs). Characterization of gene networks can shed light on the biological pathways underlying complex traits and provide the functional context for identifying those genes that contribute to disease development.

The multiple genetic, environmental, and behavioral factors that play a role in the development of alcohol use disorders (AUDs) make it difficult to identify individual genes linked to these disorders. Nevertheless, some genetic risk factors (i.e., specific variants) associated with AUDs have been identified within many genes, some of which code for proteins involved in known biological pathways. Despite this progress, it has been exceedingly difficult to determine which genes may be the most relevant to developing therapeutic interventions for alcoholism. The major obstacles in treatment development are that gene–disease associations reveal very little about the underlying biology and that any implicated gene variant explains only a tiny proportion of an individual’s overall risk for an AUD. Recent work focusing on the study of alcohol-related gene networks is helping to shed light on the molecular factors affecting alcoholism and other complex diseases. This article will provide an overview of approaches used to identify or construct gene networks and describe how systems biology approaches are helping to better understand complex traits such as behavioral responses to beverage alcohol (i.e., ethanol) and alcoholism.

## Traditional Approaches to Dissecting Complex Traits

The predominant experimental strategy used by contemporary geneticists to identify the genetic factors involved in complex traits, such as behavioral responses to alcohol, essentially is an expansion of the gene mapping approach proposed by Botstein and colleagues (1980) over 30 years ago. For this approach, investigators scan their samples for genetic variations (i.e., polymorphisms) that segregate with the trait—that is, which are found in samples with the trait more often than would be expected by chance and therefore might contribute to the development of that trait. In recent human studies, this approach typically has been applied in genome-wide association studies (GWASs) of large, population-based samples that comprise both case subjects (i.e., individuals expressing the trait, or phenotype, under investigation) and unaffected control subjects. Hundreds of complex diseases and traits, including susceptibility to AUDs, have been analyzed using GWASs, resulting in the identification of several important links between genetic variants and these diseases (Bierut et al. 2010). Overall, however, the success of this approach has been mixed, and greater progress has been hindered by insufficient sample sizes, stratified populations, the involvement of rare gene variants (i.e., alleles) that each only have a small effect size, and heterogenous phenotypic constructs (i.e., using different criteria to distinguish cases from controls).^1^

A similar forward-genetics approach that most often is used for studying animal models of complex traits is called quantitative trait locus (QTL) mapping. A quantitative trait is a phenotype that is determined by several genes, each of which has a variable contribution to the trait. The locations of the involved genes on the chromosomes are referred to as QTLs. QTL mapping studies typically are conducted using inbred strains of mice and their various derivatives. For example, the C57BL/6J (B6) and DBA2/J (D2) inbred mice frequently are used in alcohol research because they clearly differ in various responses to alcohol, including development of functional tolerance (Grieve and Littleton 1979), locomotor activation (Phillips et al. 1998), and sensitivity to withdrawal symptoms (Metten and Crabbe 1994). Because the environmental conditions in these experiments can be controlled, any differences observed between the mouse strains in these phenotypes most likely can be attributed to genetic differences. QTL mapping studies then seek to detect the polymorphisms underlying the complex traits of interest by scanning for alleles that co-vary with the traits.

Similar experiments also can be conducted with special derivatives of inbred strains known as recombinant inbred (RI) mice. These animals are derived by cross-breeding two or more distinct parental strains (which often diverge widely for the trait of interest), followed by inbreeding of the offspring for several generations (Bailey 1971). Given the correct breeding strategy, this method results in a panel of RI mouse strains that differ in the degree to which they exhibit a certain phenotype of interest. At the same time, each of the strains effectively is isogenic, meaning that for all genes, the genome carries two identical alleles (i.e., is homozygous). As a result, when two animals from the same RI mouse strain are bred, their offspring will have the exact same genetic makeup (i.e., genotype) as the parents. This makes it possible to directly integrate results generated from disparate experiments, in different laboratories, and at different times if they all use animals from the same RI mouse strain. This feature of RI mouse panels, and inbred animals in general, is particularly valuable for QTL mapping because the expense and time involved with genotyping or sequencing a strain only is incurred once.

The molecular and genetic resources outlined above have greatly increased the power and resolution of QTL mapping for various behaviors or other traits of interest. Yet despite these advances, the DNA regions identified as QTLs typically still are relatively large and may contain several genes; accordingly, few genes have been validated as contributing to quantitative traits (i.e., being quantitative trait genes [QTGs]). This difficulty is attributable largely to the lack of sufficient recombination events in existing mouse panels to reduce the size of DNA segments that typically are inherited together (i.e., haplotype block size) for fine mapping and to the generally small effect size for any single QTG.

## Genomic Approaches to Disease Dissection

Because of the technical obstacles impeding their more effective use, both GWASs and QTL mapping studies to date have identified a deluge of disease-associated genetic loci but only few actual causal genes. Moreover, even the most successful studies have failed to place the disease-associated genes in any kind of biological context that would serve to explain the underlying functional biology. Without elucidating the complex interactions of the molecular phenotypes that stand between genetic variation and disease, it will be difficult or impossible to develop new and effective approaches to treating such diseases.

The emerging field of systems biology is tackling this immense challenge by studying networks of genes, proteins, metabolites, and other biomarkers that represent models of genuine biological pathways. Studying complex diseases in terms of gene networks rather than individual genes or genomic loci should aid in uncovering disease genes. With this approach, the effects of multiple genes in the network are combined, producing a stronger signal and reducing the number of statistical tests of association that must be performed.

These benefits effectively were demonstrated in two recent human association studies that modified the typical GWASs strategy by seeking associations only within groups of functionally related genes, rather than across the entire genome. The first of these studies (Ruano et al. 2010) discovered that cognitive ability, a complex phenotype with a large genetic component, was significantly linked to genes encoding molecules called G-proteins that consist of three different subunits (i.e., heterotrimeric G-proteins). The second study (Reimers et al. 2011) found that genes related to signaling pathways involving the neurotransmitters glutamate and γ-aminobutyric acid (GABA) signaling collectively contribute to alcohol dependence.

Network-based approaches to the dissection of complex diseases also can be applied to animal models, yielding experimental results that are more generalizable to humans because the pathways represented by these networks are more evolutionarily conserved than individual genes. This should encourage greater collaboration between researchers studying a common disease in different species. In fact, the biology underlying gene networks is so complex that any hope of deriving novel therapeutics may be entirely contingent on the extent to which scientists with diverse areas of expertise are willing to share and integrate datasets and make the process of interpretation a collaborative one.

### Using High-Throughput Molecular Profiling to Define Disease

As the human and mouse genomes were being assembled using the cutting-edge, high-throughput DNA sequencers that made these endeavors possible, new technologies began to emerge that, for the first time, allowed near-comprehensive profiling of other cellular components. The term profiling refers to the measurement of different types of biological molecules, such as DNA to identify polymorphisms, messenger RNA (mRNA) to determine transcript abundance, proteins to identify certain chemical modifications that occur after the initial protein synthesis, and metabolites to evaluate biochemical processes in the cells. Platforms for high-throughput approaches for all these types of molecular profiling have become increasingly commonplace. Concurrently, methods for analyzing data produced by these technologies constantly are evolving, yielding results that are simultaneously more sensitive and more specific. As a result, researchers are better able to appreciate systems-level changes associated with disease.

Of these various high-throughput profiling techniques, microarray-based gene expression platforms have featured most prominently in biomedical research to date. Through an unbiased profiling of the transcriptome—that is, a measurement of all mRNA molecules produced within a cell or tissue sample—microarray expression studies allow researchers to identify patterns of gene expression associated with a disease. In some cases, such patterns can better define a complex phenotype by identifying disease subtypes. For example, microarray analysis of breast cancer tumors identified gene expression signatures that predict patient prognosis and therefore help physicians tailor treatment regimens (van’t Veer et al. 2002). From a basic research perspective, microarray expression profiles can help tease apart the complex interactions that underlie the development of a disease by implicating a subset of genes whose regulation is altered with the disease. With this information, it may become feasible to reconstruct the underlying biological pathways and enhance understanding of disease etiology.

Genomic approaches have been applied to alcoholism directly by studying postmortem human brain tissue isolated from alcoholics and matched control subjects using gene expression microarrays. This has revealed novel information about changes in the brain’s transcriptome that are associated with chronic ethanol consumption. One of the findings was a significant deregulation of genes encoding proteins that synthesize and maintain myelin, the substance that forms a sheath surrounding the long extensions (i.e., axons) of nerve cells and that is essential for effective nerve signal transmission (Lewohl et al. 2000; Mayfield et al. 2002). However, the nature of these studies makes it impossible to determine whether such gene expression deviations actually are risk factors that contribute to AUDs or simply represent molecular consequences of excessive alcohol consumption that are unrelated to the behaviors constituting alcoholism.

Animal models can assist greatly in this analysis by allowing for experiments that are far more detailed and informative but too invasive to ever be performed with humans. Although animal models could never replicate a phenotype as complex as alcoholism, they can mimic certain facets of the trait, which then can be associated with specific expression signatures using gene expression microarrays. For example, a genetic predisposition for alcoholism may entail a stronger-than-average preference for alcoholic beverages. This particular facet of alcoholism is captured by rodent models that selectively were bred to maximize a penchant for or aversion to ethanol, such as the aptly named high-alcohol preference (HAP) and low-alcohol preference (LAP) mice (Grahame et al. 1999). In order to identify genes that may alter the perceived desirability of ethanol, gene expression microarrays were used to compare the brain transcriptomes of HAP and LAP mice, as well as of several other inbred mouse strains that drastically differ in ethanol preference (Mulligan et al. 2006). This important study identified a diverse array of molecular pathways associated with differences in ethanol preference. Some of the genes that had the largest effect sizes were related to neuronal function and to the maintenance of the cells’ normal internal conditions (i.e., cellular homeostasis).

Another important facet of a genetic predisposition to alcoholism is a comparatively blunted sensitivity to the effects of ethanol. Studies have shown that people who initially are less sensitive to acute ethanol exposure are more likely to have a family history of alcoholism and are at greater risk for developing an AUD (Schuckit 1984, 1994). As mentioned earlier, the B6 and D2 inbred mice frequently are used in genetic studies of ethanol sensitivity. For this reason, Kerns and colleagues (2005) used microarray expression studies to dissect the effect of acute ethanol exposure on the brain’s transcriptome using the B6 and D2 inbred mouse strains. The investigators analyzed three brain regions involved in a brain system called the mesocorticolimbic reward pathway, which is involved in mediating the rewarding properties of alcohol and other drugs. For each region analyzed, the study identified a specific set of genes (i.e., a gene module) whose expression was altered in response to acute ethanol exposure. These gene modules contained greater-than-expected numbers of genes involved in several signaling pathways (i.e., retinoic acid signaling, neuropeptide expression, and glucocorticoid signaling). Moreover, similar to the microarray studies of postmortem human alcoholic brains (Lewohl et al. 2000; Mayfield et al. 2002), several genes involved in myelination robustly were altered by alcohol exposure, particularly in the prefrontal cortex (Kerns et al. 2005).

In examining the responses to acute or chronic alcohol exposure in rodent brains, these and numerous other genomic studies have enhanced the understanding of the “ethanol transcriptome” and provided a more comprehensive picture of the genes and molecular pathways that contribute to specific facets of AUDs than what is possible with studies of postmortem human brains (Daniels and Buck 2002; Mulligan et al. 2011; Rimondini et al. 2002; Saito et al. 2004; Treadwell and Singh 2004). Moreover, these studies effectively have demonstrated how gene expression microarrays can help close the information gap that exists between DNA variation and complex diseases. However, prioritizing the long lists of genes produced by comparative microarray studies conducted in either species has proven exceedingly difficult. As the costs associated with validating a given gene’s role in driving a complex trait are considerable, an effective strategy for prioritizing candidate genes is crucial. Investigators therefore have used more systems-level approaches that combine genetic, genomic, and pharmacological methods to better delineate gene networks causally related to ethanol behaviors. Networks allow us to infer relationships between genes and determine which are most important.

## The Gene Network As a Modern Genetic Map

The previous section mentioned several studies that used gene-expression microarrays to define lists of genes responding to ethanol or otherwise relevant to AUDs. Although these studies have provided important biological insights, the question of how such lists can be used to further advance understanding of a complex disease is not easily answered. Network-based approaches can greatly improve the interpretability of differential gene-expression results by providing information about the relationships between genes.

Networks are systems of interconnected components. For example, the World Wide Web is a global network of computers sharing documents connected by hyperlinks; road maps are renderings of city networks connected by highways; social networks are groups of people connected through friendships; cellular signaling pathways are groups of proteins connected through molecular interactions; et cetera. Placing such complex systems within a network framework makes it possible to formally analyze the relationships that constitute these systems. Gene networks typically are visualized as mathematical graphs—that is, a collection of vertices and edges, where genes are represented by nodes and the lines connecting the nodes indicate that some relationship exists between the genes.

Many published network analyses of gene groups use information about pre-existing biological relationships, which may be derived from sources such as literature co-citation analysis (i.e., genes mentioned together in a scientific abstract), protein–protein interaction databases, or gene ontology groupings. Some commercial tools are available for such studies, such as Ingenuity Pathway Analysis (Ingenuity Systems, Redwood City, CA). However, although these sources provide categories for interpreting the genomic data, they also force such interpretation into the mold of pre-existing information, thereby partially defeating the goal of genomic studies.

Genomic data collected with high-throughput molecular profiling presents the opportunity to derive novel gene–gene interactions. The maturity of gene expression microarrays relative to similar technologies designed to measure other molecular phenotypes on a genomic scale has meant that gene networks primarily are rendered as gene coexpression networks. In the context of gene coexpression networks, links between nodes typically indicate that the expression levels for two genes are strongly correlated with one another across whatever conditions an experiment entails (e.g., across tissues, time points, treatments, or individuals). Each link in a gene network essentially represents a testable hypothesis that can be validated through follow-up molecular experiments. Indeed, coexpression networks have been used to identify protein interactions that are novel (Scott et al. 2005) and conserved across species (Stuart et al. 2003).

Various novel and innovative methods exist for generating gene coexpression networks. Although a comprehensive review of these methods is beyond the scope of this article, a few select methods are described in more detail in the sidebar. In their simplest form, however, gene coexpression networks can be constructed by calculating Pearson correlations between all gene pairs and applying a cutoff threshold to determine which genes should be connected. The simplicity of this approach makes it an appealing choice for conducting a first round of analyses.

**Figure 1:**
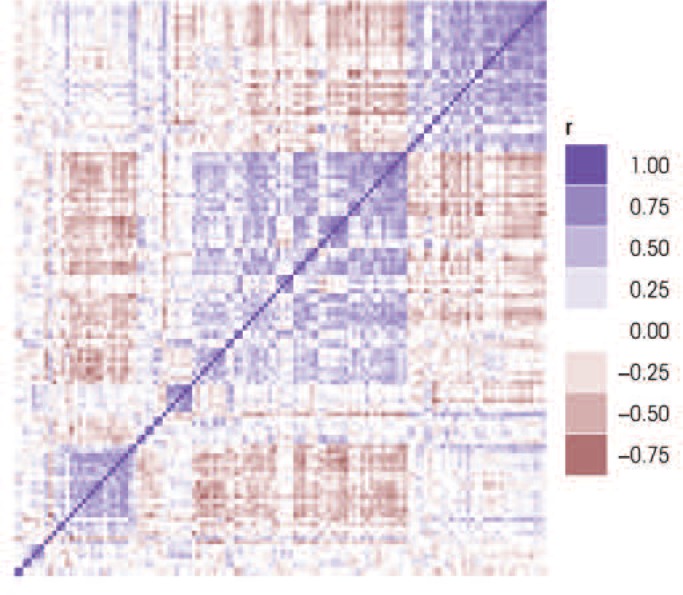
Correlation heatmap depicting patterns of co-expression among genes previously identified as being regulated by acute ethanol (Kerns et al. 2005). Each colored square represents the Pearson correlation (r) between a pair of genes, calculated using microarray expression data of prefrontal cortex tissue collected from B6, D2, and 27 BXD mouse strains. The blue and red colors indicate the strength and direction of the gene–gene correlation. Hierarchical clustering was applied to group genes based on the similarity of their expression profile across this dataset. In doing so, modules of co-expressed genes are revealed as cohesive blocks along the diagonal.

Wolen and colleagues (in press) have attempted to better define the meso-corticolimbic reward pathway’s transcriptional response to acute ethanol exposure by expanding the original B6/D2 study (Kerns et al. 2005) to include members of the BXD family of recombinant inbred mouse strains. The naturally occurring DNA polymorphisms that distinguish each BXD strain cause heritable changes in gene expression, making it possible to identify genetically coregulated transcripts across the BXD family. Microarray expression data from the prefrontal cortex of BXD family animals were used to look for evidence of coregulation among the 307 ethanol-responsive genes identified in the original B6/D2 study (see figure 1). The analysis identified several groups of intercorrelated gene modules, indicating this gene set is comprised of several gene networks (figure 1).

A variety of calculations can be used to gauge the relative importance of a particular gene to the network as a whole (Horvath and Dong 2008). The simplest measurement of node importance is determined by the degree of “connectivity”—that is, the number of other genes the node is connected to in the network. However, a gene’s “position” in the network also is an important consideration. For example, a gene that served as the sole connection between two otherwise independent gene networks would rank fairly low on a priority scale based on connectivity alone, despite being an important channel of intermodule communication. A measurement of “betweenness centrality” (Girvan and Newman, 2002) can highlight such a gene by determining the frequency with which a node is included in the shortest paths between all possible node combinations.

**Figure 2:**
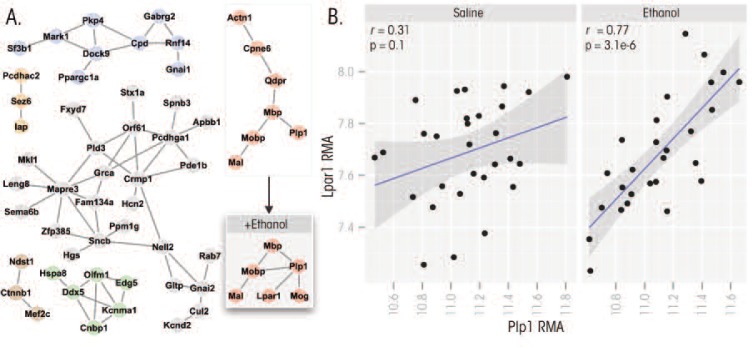
**A)** Gene networks that are regulated by acute alcohol exposure were identified in the same prefrontal cortex dataset used in Figure 1. Gene networks were generated by applying a hard threshold of 0.75 to the gene correlation matrix. The inset box contains a cognate of the myelin network (red) that was generated with expression data from the same strains following ethanol treatment. The ethanol-induced modifications of this network include the addition of a novel connection between *Plp1* and *Lpar1*. **B)** Scatterplots illustrating the correlation between *Plp1* and *Lpar1* at baseline and following ethanol treatment. The effective absence of any correlation between these genes at baseline suggests that this relationship is driven by ethanol exposure.

Figure 2 highlights six subnetworks taken from the larger coexpression network depicted in figure 1. The network comprising genes known as *Mbp*, *Mobp*, *Mal*, and *Plp1* is of particular interest, because these genes all play a role in the formation and stabilization of myelin. In addition, a parallel analysis was conducted using microarray data only from the prefrontal cortex of the same BXD strains after they had received an injection of 1.8 g/kg ethanol into their abdominal cavity. This analysis revealed that the myelin gene network persisted but underwent minor topographical modifications. Most notably, additional connections were detected between *Plp1* and two additional genes called *Mog* and *Lpar1*. The absence of *Mog* in the original network probably was an artifact of the method used to form these networks, and the gene likely should have been included. *Plp1* and *Lpar1*, in contrast, were effectively unrelated at baseline and only showed evidence of coregulation after ethanol treatment, suggesting this is a genuine molecular response to ethanol (see figure 2B).

The *Lpar1* gene encodes a receptor for lysophosphatidic acid (LPA), a signaling molecule containing phosphate and lipid components (i.e., a phospholipid). Regulation of *Lpar1* is critical for proper nerve-cell formation (i.e., neurogenesis), including in a brain region called the hippocampus in adults (Matas-Rico et al. 2008). In addition, *Lpar1* regulates the breakdown of the myelin sheath (i.e., demyelination) that occurs after nerve injury (Inoue *Lpar1* regulates the breakdown of the myelin sheath (i.e., demyelination) that occurs after nerve injury (Inoue et al. 2004). The fact that *Lpar1* is brought into this network by ethanol exposure suggests the intriguing possibility that this gene may play a role in the loss of white matter^2^ commonly observed in long-term alcoholic patients (Kril and Halliday 1999). This example illustrates how studying ethanol-induced changes in gene-network topology can produce testable hypotheses relevant to the neurobiology of alcoholism. Obviously, alterations in the gene network occurring after acute ethanol exposure might not always be relevant to alterations in brain structure and function (i.e., neural plasticity) or toxic effects that occur with chronic exposure, such as in alcoholism. Therefore, findings regarding networks relevant to one ethanol behavioral phenotype should be considered “specific” to that phenotype unless other genetic, pharmacological, or behavioral data suggests links to other aspects of ethanol’s actions in animal models or humans. More generally, this example demonstrates how systems-level methods, like gene coexpression analysis, can help greatly expand the information content of gene expression microarray studies by filling in information about the gene–gene relationships.

**Constructing Gene Networks**

Various methods exist for generating gene networks. As mentioned in the main text, the simplest method for constructing gene coexpression networks involves calculating Pearson correlations for all pair-wise genes and applying a hard threshold to determine which genes should be connected. The robustness of these networks, initially referred to as “relevance networks,” can be assessed through an approach called permutation testing (Butte et al. 2000). A more rigorous method for constructing gene coexpression networks utilizes a graph theoretical approach to identify densely intercorrelated gene modules called paracliques (Baldwin et al. 2005). Paracliques represent gene–gene interaction networks with extensive, but not perfect, strong expression correlations between all genes in the network (http://grappa.eecs.utk.edu/grappa/root). Paracliques can contain members with missing links. Therefore, paracliques provide an attractive compromise by augmenting coexpression with genes whose correlational relationships to a network are strong, but permissibly imperfect with a proportion of the network. This proportion, called the proportional glom factor, is a user-defined parameter.

A potential limitation of both relevance networks and paracliques is that they rely on hard thresholds to classify the relationship between genes as either connected or unconnected. The dichotomy imposed by this approach may be artificially limiting these networks, causing biologically meaningful relationships to be overlooked (Carter et al. 2004). For example, the absence of the *Mog* gene from the myelin network described in the main article and depicted in figure 2A following ethanol treatment is symptomatic of this limitation. An approach called weighted gene coexpression network analysis (WGCNA) is an increasingly popular method that avoids these potential pitfalls by utilizing a “soft-thresholding” approach to generate networks that conform to a scale-free topology (Zhang and Horvath 2005). Scale-free networks follow the power distribution they are named for, comprising many nodes that have sparse connections and a few that are highly interconnected. In addition to providing an accurate model for metabolic networks (Jeong et al. 2000), neural networks of the roundworm *Caenorhabditis elegans* (Watts and Strogatz 1998), and the World Wide Web (Albert and Jeong 1999), the scale-free topology also typifies gene coexpression networks (van Noort et al. 2004). Some researchers recently have used WGCNA to define correlated gene modules associated with blood alcohol levels using the “drinking-in-the-dark” paradigm of excessive ethanol consumption in B6 mice (Mulligan et al. 2011). WGCNA also can be implemented as a freely available package (Langfelder and Horvath 2008) for the R Statistical Environment and provides an excellent set of tutorials (available at genetics.ucla. edu/labs/horvath/CoexpressionNetwork/ Rpackages/WGCNA).

## Bridging the Gap Between Genomics and Gene Mapping

### Genetical Genomics

Another important early advancement toward a more systems-level approach to identifying disease-associated genes was the application of gene mapping methods to high-throughput molecular data in order to identify causal links between molecular phenotypes and genomic regions. Like classical physiological or behavioral phenotypes, genetic factors influencing high-throughput measures of transcript, protein, and metabolite abundance can be identified by QTL mapping. To date, such analyses mostly have been applied to gene-expression microarrays, mapping gene expression QTLs (eQTLs). This largely is related to technical constraints, because whole-proteome expression profiling currently cannot be done with the same degree of sensitivity, coverage, and throughput as mRNA profiling.

The strategy of performing genetic linkage analysis on genome-wide molecular profiles was formalized and termed “genetical genomics” by Jansen and Nap (2001). This proposal primarily focused on gene-expression microarrays and posited that mapping eQTLs would enable researchers to construct robust gene networks as well as link these networks to metabolic or other phenotypes. The investigators also suggested that eQTL mapping could greatly aid in the identification of candidate genes underlying classical QTLs for disease traits. The first study to carry out QTL analysis across genome-wide gene expression microarrays was conducted using an experimental cross between two strains of the yeast *Saccharomyces cerevisiae* (Brem et al. 2002). Subsequently, several investigations applied the approach to mammalian systems (Schadt et al. 2003; York et al. 2005), including brain gene expression (Chesler et al. 2003, 2005).

**Figure 3:**
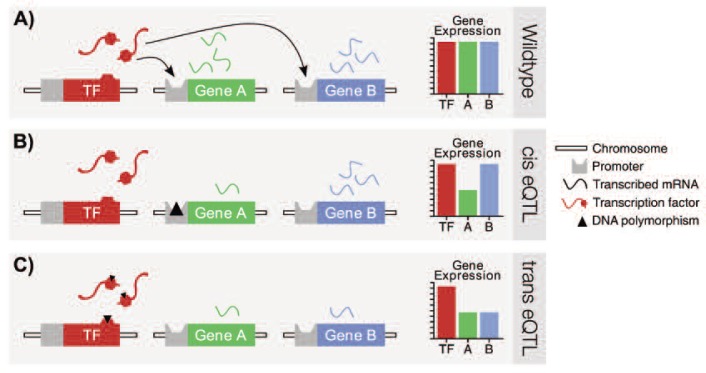
Illustration of the concept of *cis* and *trans* expression quantitative trait loci (eQTLs). **A)** The left-most gene (red) codes for a transcription factor (TF) protein that activates the transcription of genes A (green) and B (blue) by binding to their respective promoters (gray). In the wildtype, or “normal,” scenario all genes are transcribed at their full potential, as indicated by the bar graph on the right. **B)** A variant (i.e., polymorphism) (triangle) in gene A’s promoter region hinders TF binding, causing a reduction in the rate at which gene A is transcribed, while gene B is unaffected. Thus, gene A is being regulated by a *cis* eQTL because its level of expression is associated with a nearby polymorphism located on the same chromosome. **C)** A polymorphism in the TF gene’s DNA binding region (hexagon)—the region of the TF protein that binds to gene promoters—hinders binding with all downstream promoters, regardless of whether the regulated gene is located near the TF gene, like gene A, or located on an entirely different chromosome, like gene B. In fact, all genes regulated by this TF would be linked to a *trans* eQTL at the site of this TF polymorphism.

These early genetical genomics studies also characterized the two major classes of eQTLs, labeled *cis* and *trans* eQTLs, which differ with respect to the position of the eQTL relative to the gene whose expression is altered (figure 3).

A *cis* eQTL is located at the same site of the genome as the gene under study. In contrast, a *trans* eQTL can be located elsewhere in the genome, away from the gene whose expression is altered. A good example of how a *trans* eQTL could manifest involves transcription factors (TFs). These are proteins that bind with regulatory DNA regions that are located in front of a gene. Only when the TF binds to the corresponding DNA sequence can the first step in the process of gene expression—transcription—begin. The interaction between the TF and the DNA involves a certain part of the TF called the TF DNA–binding domain that allows the TF to recognize and bind with specific regulatory DNA sequences. Through this mechanism, certain TFs only may activate the transcription of specific sets of genes. Accordingly, a polymorphism at the DNA-binding domain of a certain TF can affect the TF’s ability to recognize and bind its recognition sites, causing altered expression of all genes regulated by this TF. In other words, the abundance of all transcripts from those genes would co-vary with the TF polymorphism. Such a case might be recognized by a clustering of *trans* eQTLs at the site of the causal polymorphism, sometimes referred to as a regulatory hotspot. The identification of *trans* eQTL clusters can be a powerful approach for identifying key regulators underlying a complex trait of interest.

**Figure 4:**
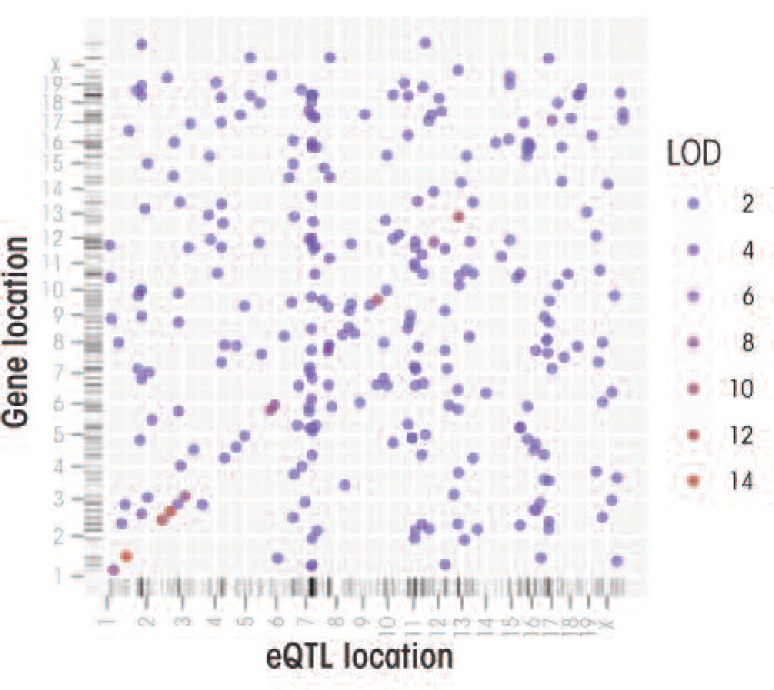
Genome–genome plot of peak expression quantitative trait loci (eQTLs) for the same dataset used in figure 1. Each point indicates the chromosomal position of a gene versus the position of its peak eQTL. Point color is used to communicate the strength of association between a gene and its eQTL, measured by logarithm of the odds (LOD) score. A LOD score is a ratio that measures that probability that a gene is linked to genetic markers, versus the probability that it is not. Thus, the higher a LOD score, the more likely a gene’s expression level genuinely is regulated by an eQTL. Points plotted along the diagonal likely represent *cis* eQTLs, which also tend to have stronger LOD scores. Perpendicular tick-marks along both axes show the distribution of data points. Along the x-axis the dense clustering of tick-marks toward the proximal tip of chromosome 7 indicates the presence of a *trans* eQTL cluster, suggesting this region may harbor an important regulator of the gene co-expression modules seen in figure 1.

Figure 4 depicts the eQTL results for the same list of 307 ethanol-responsive genes identified in the B6/D2 study that earlier was used to construct coexpression networks. This analysis revealed that these coexpression networks share common eQTLs that drive this coordinated expression. Furthermore, the strongest eQTLs underlying many of these genes mapped to one end of chromosome 7, forming a *trans* eQTL cluster. These findings provide preliminary evidence that acute ethanol–responsive genes comprise a handful of gene coexpression networks in the pre-frontal cortex and that a key regulator of these networks resides on chromosome 7. A more extensive analysis of this type has recently been completed (Wolen et al., in press).

The genes comprising *trans* eQTL clusters often have biological functions that have been conserved among species, suggesting that these hotspots may have a biological relevance. Accordingly, the search for *trans* eQTLs may allow researchers to identify biological functions associated with complex traits through defining quantitative trait gene networks (QTGNs). Mozhui and colleagues (2008) have, for example, dissected a *trans* eQTL cluster on distal mouse chromosome 1 and identified a candidate gene (*Fmn2*) that they propose has a major influence on the expression of linked gene networks. Moreover, a diverse group of phenotypic QTLs seemed to be located in the same region, including several related to ethanol.

### Genetical Genomics Studies to Identify Gene Variants Increasing Disease Risk

The integration of eQTL and classical QTL data enables identification of key markers of disease-causing variants. The effectiveness of this approach was demonstrated by a genetical genomics analysis of liver expression data from a population of mice placed on a high-fat diet (Schadt et al. 2003). The purpose of this diet was to model an obesity-like phenotype, which was measured using fat-pad mass (FPM). QTL mapping for FPM revealed a significant QTL on chromosome 2 that also harbored over 400 eQTLs. By scanning this region for *cis* eQTL-linked genes that also were strongly correlated with FPM, the researchers were able to identify two novel obesity candidate genes.

Saba and colleagues (2006) used a similar approach to identify candidate genes for alcohol preference and acute functional tolerance to alcohol. This large-scale study included rodent strains selectively bred for ethanol phenotypes (i.e., HAP and LAP mice) as well as a subset of the BXD family of recombinant inbred mice. Applying microarray expression profiles using mRNAs obtained from the entire brain, the investigators identified independent lists of genes whose expression differed between the HAP and LAP strains and between the BXD strains with high and low levels of acute functional tolerance. The genetic regulation of these gene lists then was mapped using BXD expression and genotypic data. High-priority candidate genes were high-lighted by screening for differentially expressed genes with *cis* eQTLs that overlapped previously mapped behavioral QTLs for either alcohol preference (Belknap and Atkins 2001) or acute functional tolerance (Kirstein et al. 2002).

The rationale for prioritizing candidate QTGs on the basis of their having *cis* eQTLs located at the same sites as classical QTLs is based on the hypothesis that the variability of a complex phenotype is linked to a particular locus because the causal gene is being produced in variable quantities through a *cis-*acting polymorphism. This hypothesis is somewhat of an oversimplification and leaves out several important caveats. Nevertheless, increasing evidence supports the importance of gene-expression variability in regulating complex traits. In fact, recent evidence indicates that SNPs associated with a variety of complex traits are more likely to contain *cis* eQTLs than normally would be expected (Nicolae et al. 2010). This indicates that the importance of expression variability in complex trait regulation is not limited to genetic model systems and that it may be possible for GWASs and QTL mapping studies to improve their track record by incorporating expression data.

### Dissecting Complex Diseases Through Integrating Genomic Approaches

**Figure 5:**
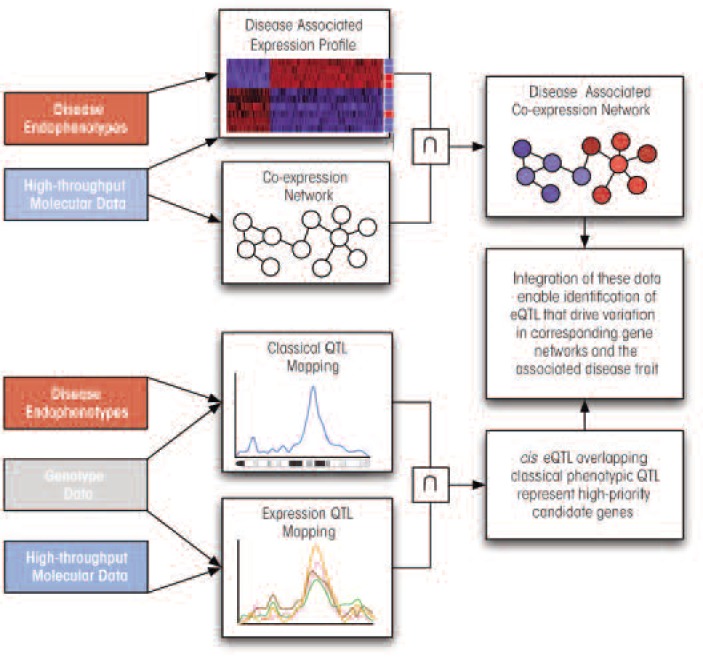
Diagram of how the genomic approaches discussed here can be integrated to identify gene networks and candidate genes for complex traits such as alcoholism. The information flow indicates how gene networks, expression quantitative trait locus (eQTL) and behavioral QTL analyses can be used together to identify candidate genes as potential targets for intervention. Note that resulting networks or candidate genes are entirely derived from experiments rather than relying on prior knowledge. In some cases, use of biomedical literature on gene–gene interactions can be used to augment such experimentally-derived networks.

The discussion above has illustrated how traditional QTL mapping and GWASs approaches can benefit from systems-biological approaches by filling in critical information about the molecular phenotypes that stand between DNA variation and complex disease (figure 5). The incorporation of data from high-throughput molecular profiling technologies, such as gene expression microarrays, can better define a disease by identifying groups of genes that respond to or covary with disease-associated traits. Network analysis of disease-associated genes allows investigators to move beyond static gene lists, partially reconstruct the underlying molecular pathways, and prioritize genes based on their importance to the larger network. Applying QTL mapping to each gene’s expression trait makes it possible to identify the genomic regions that regulate each gene’s expression and uncover the existence of regulatory hotspots that exert enormous influence over a gene network. The series of studies discussed below has demonstrated how effective these methods are for dissecting complex traits, particularly when they are integrated.

Zhu and colleagues (2004) followed up the genetical genomics analysis of liver expression data from mice on a high-fat diet that was mentioned earlier (Schadt et al. 2003) by generating gene networks from the same microarray gene-expression dataset. This analysis included two distinct approaches to network construction: The first strategy formed networks on the basis of the covariation among gene-expression traits—that is, genes whose expression changed in the same manner were considered linked. In the second strategy, gene-network interactions were determined on the basis of the similarity of their eQTL profiles. Thus, the networks were constructed once without and once with the benefit of genotypic and eQTL data. Because links within gene networks represent causal relationships, analyses of these links can help researchers predict how a system will respond to the perturbation of a specific gene. Zhu and colleagues (2004) tested this hypothesis by measuring differential expression in response to a pharmacological substance that interfered with the function of a central gene in both predicted networks. They found that the eQTL profile network was a significantly better predictor of which genes would be affected by the pharmacological perturbation than the network constructed with expression data alone. Therefore, integrating both gene-expression and genotypic information into network construction greatly enhances the predictive value of gene networks.

The ultimate goal of systems-level analyses of complex diseases is to uncover information necessary to establish a correlation between disease phenotype, mRNA abundance, and the underlying DNA polymorphism or causal gene network. Schadt and colleagues (2005) demonstrated this approach of integrating genotypic data, gene-expression data, and disease endophenotypes,^3^ using the same liver expression dataset mentioned above, and a novel network construction technique termed likelihood-based causality model selection (LCMS). The investigators first identified all QTLs associated with a classical phenotype and then winnowed the list of potentially associated gene-expression traits on the basis of their correlation or eQTL overlap with the phenotype of interest. Candidate genes then were ranked by applying the LCMS technique, which uses the eQTL data to establish causal relationships between DNA loci and transcripts as well as between transcripts and phenotypes and finally identifies a model that best fits the data.

By ranking genes according to their performance in these models, the investigators identified several novel obesity candidate genes as well as uncovered additional support for the involvement of a gene called *Hsd11b1* that previously had been implicated in obesity risk (Rask et al. 2002). Because this gene seemed to be relevant to the phenotype they were investigating, the researchers then sought to reconstruct the gene network in which *Hsd11b1* participates by performing the LCMS procedure with *Hsd11b1* as the trait of interest. The resulting network was able to successfully predict genes that would be affected by inhibition of *Hsd11b1*. A similar approach has been used by other investigators to identify transcriptional networks associated with ethanol sensitivity behavior in the fruit fly *Drosophila melanogaster* (Morozova et al. 2011). This progression from phenotype to gene network to candidate gene and back to a gene network is a striking example of the promise that combining genetical genomics and gene-network analysis provides for understanding complex traits such as alcoholism.

As previously mentioned, such network-based techniques also have been applied to provide novel insight into the functional consequences associated with ethanol exposure in the mesocorticolimbic reward pathway. Preliminary results have identified several gene-coexpression networks that are robustly altered by ethanol in a tightly coordinated fashion (Wolen et al., in press). These studies used the BXD panel, so that the genetic regulation of ethanol-induced expression changes and behavioral responses also could be examined. Similar to results shown in figure 1, this analysis has revealed that ethanol-responsive gene networks are regulated by a small number of loci that largely are specific to a given network. At least one of these loci overlaps a previously mapped QTL for loss of righting reflex, a measure of acute ethanol sensitivity (Bennett et al. 2002; Markel et al. 1997). This work suggests that focusing on identifying gene networks both greatly reduces the complexity of whole-genome expression data and provides a wealth of hypotheses regarding both functional implications and regulatory mechanisms relevant to ethanol’s action. expression profiles associated with ethanol or alcoholism has provided modern neuroscience with a wealth of molecular information regarding ethanol’s effects on the body. At the same time, alcohol researchers must make sense of a plethora of weak genetic signals and large lists of genes whose expression changes in response to alcohol. Newer approaches, such as exome^4^ sequencing studies and certain approaches to analyzing gene expression (e.g., RNA-Seq analyses), promise added clarity but also may deliver even more confusing data. By combining behavioral, genetic, and genomic studies through genetical genomics and gene-network analysis designs, researchers may be able to construct gene networks rich in functional relations to ethanol behaviors. Additional refinements in ethanol-related gene-network structures and causal relation of such networks to aspects of ethanol-induced behaviors will provide a new generation of candidate genes for therapeutic intervention in alcoholism.

## Footnotes

1 This is an issue faced by GWASs researchers when classifying samples as cases or controls. If cases are limited to only individuals who have been diagnosed with an AUD, it becomes difficult to enlist a sufficient number of participants. Moreover, many of the control subjects could very well be undiagnosed alcoholics or people who meet some but not all of the diagnostic criteria for an AUD. As a result, the control group could be polluted with near-cases, diluting any detectable group differences.

2 The term “white matter” refers to brain structures made up primarily of nerve fibers that are enclosed by the myelin sheaths and therefore have a whitish appearance. Conversely, the term “gray matter” refers to brain structures composed mainly of the bodies of nerve cells, which have no myelin sheath, resulting in a grayish appearance.

3 An endophenotype is a heritable trait or characteristic that is thought to be an intermediate phenotype between a genetic predisposition and a clinical disorder; for example, certain neurobiological characteristics have been noted in people with alcoholism and may be used as endophenotypes to identify people at risk for alcoholism. Endophenotypes are thought to be useful for gene identification under the assumption that they are simpler and closer to the genetic underpinnings of the disorder.

4 The exome is the part of the genome formed by the coding portions of genes (i.e., exons) that provide the genetic blueprint used in the synthesis of proteins and other functional gene products. It is the functionally most relevant part of the genome and, therefore, most likely to contribute to the phenotype of an organism.
